# Absorption of pressurized methane in normal and supercooled *p*-xylene revealed via high-resolution neutron imaging

**DOI:** 10.1038/s41598-022-27142-6

**Published:** 2023-01-04

**Authors:** Ondřej Vopička, Tereza-Markéta Durďáková, Petr Číhal, Pierre Boillat, Pavel Trtik

**Affiliations:** 1 Department of Physical Chemistry, University of Chemistry and Technology, Prague, Technická 5, 166 28 Prague 6, Czech Republic; 2 Laboratory for Neutron Scattering and Imaging, Paul Scherrer Institut, 5232 Villigen PSI, Switzerland; 3 Electrochemistry Laboratory, Paul Scherrer Institut, 5232 Villigen PSI, Switzerland

**Keywords:** Thermodynamics, Imaging techniques, Phase transitions and critical phenomena

## Abstract

Supercooling of liquids leads to peculiarities which are scarcely studied under high-pressure conditions. Here, we report the surface tension, solubility, diffusivity, and partial molar volume for normal and supercooled liquid solutions of methane with *p*-xylene. Liquid bodies of perdeuterated *p*-xylene (*p*-C_8_D_10_), and, for comparison, *o*-xylene (*o*-C_8_D_10_), were exposed to pressurized methane (CH_4_, up to 101 bar) at temperatures ranging 7.0–30.0 °C and observed at high spatial resolution (pixel size 20.3 μm) using a non-tactile neutron imaging method. Supercooling led to the increase of diffusivity and partial molar volume of methane. Solubility and surface tension were insensitive to supercooling, the latter substantially depended on methane pressure. Overall, neutron imaging enabled to reveal and quantify multiple phenomena occurring in supercooled liquid *p*-xylene solutions of methane under pressures relevant to the freeze-out in the production of liquefied natural gas.

## Introduction

Liquids cooled below their melting points show peculiar physical properties, such as changes in viscosity and diffusivity, which are commonly ascribed to the dynamic heterogeneity on the molecular level^1–6^. Thermal movements of molecules slow down either due to energy barriers or lack of free volume^1,7–9^. Specific interactions, such as hydrogen bonds and interactions among aromatic rings, can be involved. For instance, the formation of ice-like clusters in supercooled water was hypothesized since organic compounds, such as *o*-, *m-*, *p*-xylene, are volatilized (Henry’s constants increase) from their supercooled solutions with water^10^. Besides water, specific interactions likely influence the properties of other supercooled liquids, such as *p*-xylene. This compound, which solidifies at 0.84 GPa at room temperature, was reported to form trimers and tetramers at 13.5 GPa^11^. Thus, the molecular-level heterogeneity of supercooled liquid *p*-xylene can be expected to occur and to be involved in the spontaneous solidification and hardly predictable properties due to the thermodynamic instability.

The high normal melting point of *p*-xylene (*p*-C_8_H_10_, 13.25 °C^12^) causes that this compound can condense, become supercooled and deposit on cold spots in the production of liquefied natural gas (LNG). The other xylene isomers are much less severe, the normal melting points^12^ are: − 25.17 °C (*o*-C_8_H_10_) and − 47.85 °C (*m*-C_8_H_10_). Besides that, *p*-xylene is a practical test compound other than water for studying supercooled liquids at rather mild temperatures. Although condensation of the BTEX compounds (benzene, toluene, ethylbenzene, xylenes) in the production of LNG is avoided by their low admitted concentration (< 1 ppm^13^), this study can bring a better understanding of phenomena occurring on cold spots in the processing of natural gas and broaden the general knowledge.

Recent studies have reported vital experimental data and predictive models describing compositions of coexisting phases^13,14^ and, importantly, the melting temperature for *p*-xylene (*p*-C_8_H_10_) in systems comprising of methane (CH_4_) and *p*-xylene (*p*-C_8_H_10_)^14^. Thus, the degree of supercooling can be assessed not only for pure *p*-xylene but also for its mixtures with methane at elevated pressures. The achievable supercooling generally depends on the chemical composition and surfaces in the experimental device. The supercooling by about 2 °C was reported to induce *p*-xylene solidification at a cooled copper tip for methane (CH_4_) and *p*-xylene (*p*-C_8_H_10_) solution at 4.35 °C and 225 bar^14^. In contrast to the literature^14^, in which solidification was induced, we aim at studying the properties of the thermodynamically unstable supercooled liquid.

Experimental data on density, solubility, speed of sound, heat capacity, surface tension and viscosity have been so far reported for several supercooled liquids, chiefly water^3,10,15–29^. With the exception of one investigation on the speed of sound and derived quantities for supercooled water^16^, the above studies report data observed at pressures near atmospheric or do not report experimental pressure. The common high-throughput methods for studying liquids under high-pressure conditions are the vibrating tube densimetry, pendant drop method, Taylor dispersion method, the method of capillary waves, methods utilizing Raman spectroscopy and Nuclear Magnetic Resonance^30–41^. To our knowledge, no report on their use for supercooled liquids under high pressures is available. We certainly admit that these or other methods can be applied to study the properties of supercooled liquids. For instance, Raman spectroscopy and Nuclear Magnetic Resonance were used for studies on the formation of natural gas hydrate under relevant conditions^42,43^. As we show in this study, our non-tactile one-pot neutron imaging method^44^ is applicable for studying systems involving supercooled liquids exposed to pressurized gases.

In this work, methane (CH_4_) absorption in liquid perdeuterated *p*-xylene (*p*-C_8_D_10_) and *o*-xylene (*o*-C_8_D_10_) was studied while this choice of isotopic composition exploits the high neutronic contrast^45^ between protium (H) and deuterium (D). The influence of the isotopic composition on the physical properties of chemical species has been thoroughly reported for benzene rather than for the xylenes and appears low. For instance, the molar volume (molar mass over density) of perdeuterated benzene (C_6_D_6_) differs by less than 0.24% from that of benzene (C_6_H_6_) under conditions relevant for this study^46^; see Supplementary Information (SI) for more discussion. Viscosity, melting point, boiling point and surface tension of perdeuterated benzene differ from those of benzene by 5%^47^, 1.0 °C^48^, 0.8 K^49^ and 2% (− 0.5 mN·m^−1^)^50^, respectively. Systematical errors caused by using deuterated xylenes instead of the protium-based (normal) ones are expected to be comparable to those for benzene.

## Results and discussion

### Composition revealed using neutron imaging

Liquids were maintained at a constant temperature and exposed to a methane pressure step at the zero time in cylindrical cells^44^. Since the cells were axially symmetric (inner diameter 9.0 ± 0.1 mm), the onion-peeling algorithm^51^ was used to provide the tomographic reconstructions at the central plane of the sample. The overall linear attenuation coefficient of the liquid (*Σ*) was contributed by the two components, A (CH_4_) and B (*p*- or *o*-C_8_D_10_). The Beer-Lambert law thus has the form

1 $$\ln \frac{{I^{0} }}{I} = {\upsigma }_{{\text{A}}} N_{0} c_{{\text{A}}} d + {\upsigma }_{{\text{B}}} N_{0} c_{{\text{B}}} d = \Sigma_{{\text{A}}} d + \Sigma_{{\text{B}}} d$$

**Figure 1:**
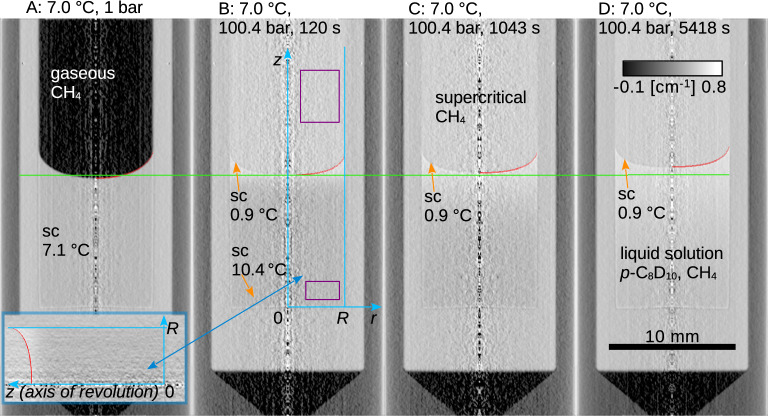
Tomographic reconstructions of the measuring cell with supercooled perdeuterated *p*-xylene (*p*-C_8_D_10_) exposed to methane at 1.0 bar (**A**) and at 100.4 ± 0.2 bar at 7.0 ± 0.2 °C (**A**)–(**D**). Time after exposure to pressurized methane, fits of the phase interface shapes (red curves), local level of supercooling (sc), axes and cell radial dimension (blue lines, 2·*R* = 9.0 ± 0.1 mm), and domains used for the calculation of the linear attenuation coefficient of the pure components (purple boxes) are indicated. Green dashed line is the liquid level at the cell center short after the pressurization. The bottom-left insert shows the body of revolution used for the calculation of the liquid volume.

Cross-sections (σ) of the pure components were evaluated based on the tomographic reconstruction observed just after the release of pressurized methane to the vessel with sample liquid by assuming negligible evaporation of the liquid and diffusion of methane to the bottom part of the liquid body (Fig. 1). Symbols *I*, *N*_0_ and *d* are intensity, Avogadro number and length, respectively. The mole concentration (*c*) and density ($\rho$) of pure methane in the gas and supercritical fluid phase were calculated using the Peng-Robinson equation of state^52^. Evaporation of the xylenes was neglected^53,54^; see SI for the assessment of the combined systematic uncertainty. For the liquid perdeuterated xylenes, mole concentration and density were calculated from the known equations of state for the pure *p*- and *o*-xylene (*p*- and *o*-C_8_H_10_) liquids^46^ by assuming that the molar volume of the deuterated and protium-based chemicals are equal (see SI for uncertainty estimation). Cross-sections and densities for the pure components at the studied conditions are listed in Table S1 in SI. In the case of supercooled *p*-xylene, density was calculated by extrapolating from the region of (normal) liquid. Since the cross-section of *p-*xylene held constant over all the inspected conditions, such extrapolation provided a meaningful approximation and supercooled *p*-xylene did not solidify as the solidification of *p-*xylene is accompanied with the change of density by about 20%^12^. The level of supercooling was assessed as the difference between the melting temperature and the actual temperature using the available literature data for the melting point of the protium-based *p*-xylene (*p*-C_8_H_10_) under relevant conditions, see SI and *Materials and Methods*. It is substantial to note that the liquid swelled during methane absorption, which did not enable for the simplistic use of Beer-Lambert law, Eq. (1).

### Phase interface, swelling, diffusion

The shape of the mobile axially symmetric phase interface in gravity (red curves in Fig. 1) was parameterized by solving the Young–Laplace equation in form^55,56^

2 $$z = \frac{\gamma }{\Delta \rho \cdot g}\left( {\frac{{z^{^{\prime\prime}} }}{{ (1 + z^{^{\prime}2})^{3/2} }} + \frac{{z^{^{\prime}} }}{{r\left( {1 + z^{^{\prime}2} } \right)^{1/2} }}} \right)$$

The boundary value problem, *z'*(*r* = 0) = 0 and *z'*(*r* = *R*) = cot(θ), was solved using the *midrich* method as in the Maple 2021 software package, *r* ∈ (0, *R*). Optimum values of the surface tension (γ) and contact angle at the wall of the cell (θ), and their uncertainty due to random errors (*u*_r_, cover factor 2) were calculated using Gauss–Newton and Bonferroni methods^57,58^, combined systematic uncertainty of surface tension was calculated using the law of uncertainty propagation (see SI). The density difference at the phase interface, ∆*ρ*, was calculated as follows.

**Figure 2:**
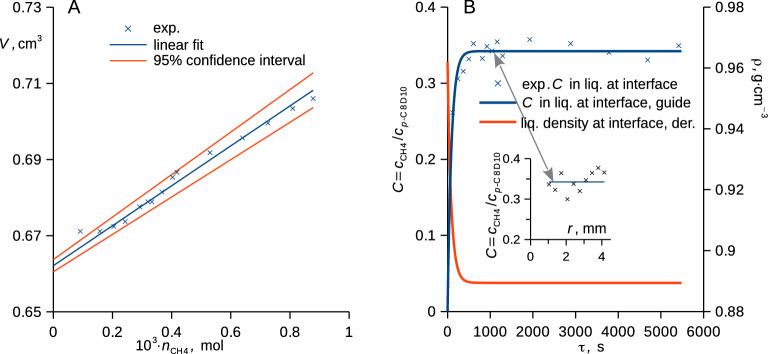
(**A**) Plot of volume of the liquid in the cell against the mole amount of absorbed methane is shown together with the linear fit and its confidence interval. (**B**) Time-evolution of the measured radius-averaged relative concentration of methane at the phase interface is shown with an empirical model (the blue curve is the plot of *C*_∞_ − *C*_∞_⋅exp[− τ / (91.1 s)]). The red curve is the derived model of density, insert shows the relative concentration of methane at the interface with respect to the cell radius for time 1043 s. Both examples are for supercooled perdeuterated *p*-xylene (*p*-C_8_D_10_) at 7.0 ± 0.2 °C initially exposed to methane at 100.4 ± 0.2 bar.

The volume of the liquid (*V*) was calculated by computing the volume of the solid of revolution (see insert in Fig. 1), which is a generalization of an earlier method utilizing projections (photography in visible light) of phase interfaces in glass tubes without using the reconstruction of the central plane^59^. The partial molar volume of methane ($\overline{V}_{{\text{A}}} = \partial V/\partial n_{{\text{A}}}$) and its uncertainty due to random errors (*u*_r_, cover factor 2) were calculated based on the mole amount of absorbed methane ($n_{{\text{A}}}$) in the entire liquid body and its volume at fixed *T*, *p*, and mole amount of the perdeuterated xylene ($n_{{\text{B}}}$), see Fig. 2A and Table S4 in SI. The molar volume ($V_{{\text{m}}}$) of the liquid and its density ($\rho$) depend on the mole fractions [$x_{{\text{A}}} = n_{{\text{A}}} /\left( {n_{{\text{A}}} + n_{{\text{B}}} } \right)$_,_ $x_{{\text{B}}} = 1 - x_{{\text{A}}}$] and molar masses (*M*) such that

3 $$V_{{\text{m}}} = \left( {x_{{\text{A}}} M_{{\text{A}}} + x_{{\text{B}}} M_{{\text{B}}} } \right)/\rho = x_{{\text{A}}} \overline{V}_{{\text{A}}} + x_{{\text{B}}} \overline{V}_{{\text{B}}}$$

where the partial molar volume of each perdeuterated liquid xylene was set to its molar volume.

**Figure 3:**
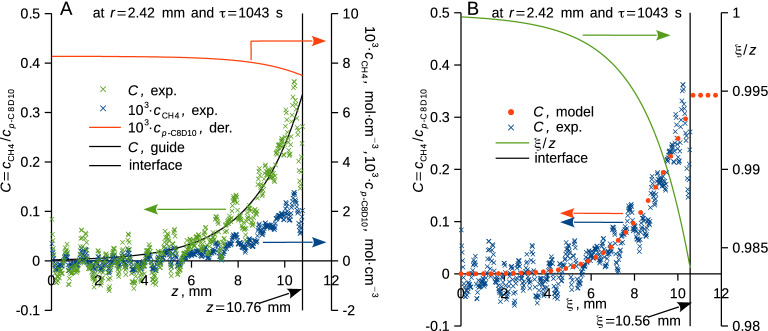
(**A**) Experimental relative (*C*) and mole (*c*) concentrations of methane, and the derived mole concentration of *p*-xylene (orange curve) are plotted against the depth coordinate (*z*) together with a guide for the eye (black curve) used for the extrapolation to the interface. (**B**) Experimental (blue crosses) and modelled (orange points, Fick’s second law) relative concentrations of methane are plotted against the B-fixed depth coordinate ($\xi$) together with the $\xi /z$ ratio (green curve). Phase interface is indicated by vertical dashed lines, both figures are for supercooled perdeuterated *p*-xylene (*p*-C_8_D_10_) at 7.0 ± 0.2 °C initially exposed to methane at 100.4 ± 0.2 bar, radius coordinate and time upon pressurization are indicated.

The simultaneous measurement of the neutron attenuation and the shape of the liquid (and thus volume) enabled to derive the concentration distributions of both species (Fig. 3A) using Eqs. (1) and (3), constant mole amount of the xylene in the liquid was assumed. This, in turn, enabled to extrapolate the methane concentration in the xylene phase at the phase interface and thus to derive the respective density (Figs. 2B, 3A) needed for the Young–Laplace equation, Eq. (2). Moreover, the *z* coordinate (depicted in Fig. 1B) was transformed to the B-fixed coordinate $\xi$ (Fig. 3B) along which mole concentration of the perdeuterated xylene (${c}_{\mathrm{B}}$) is fixed.

Diffusion of methane in the axially symmetric liquid body (Fig. 1) was modelled using Fick’s second law in cylindrical B-fixed coordinates in form^60^

4 $$\frac{\partial c}{{\partial \tau }} = D\frac{{\partial^{2} C}}{{\partial \xi^{2} }} + D\frac{1}{r}\frac{\partial }{\partial r}\left( {r\frac{\partial C}{{\partial r}}} \right)$$

where *D* is diffusivity in the B-fixed reference frame and $\xi$ ranges from zero to the initial liquid level at a given radius. Concentration at the phase interface (Fig. 2B) and its shape (Fig. 1) were used for the construction of the Dirichlet boundary condition; impermeable walls of the cell were represented by Neumann boundary conditions. We have solved Eq. (4) using an explicit differentiation scheme^58^, and calculated optimum value and uncertainty due to random errors (*u*_r_, cover factor 2) of diffusivity (*D*) using Gauss–Newton and Bonferroni methods^57,58^. Fick’s second law, Eq. (4), provided a good approximation of the experimental data (Fig. 3B).

### Surface tension and solubility

**Figure 4:**
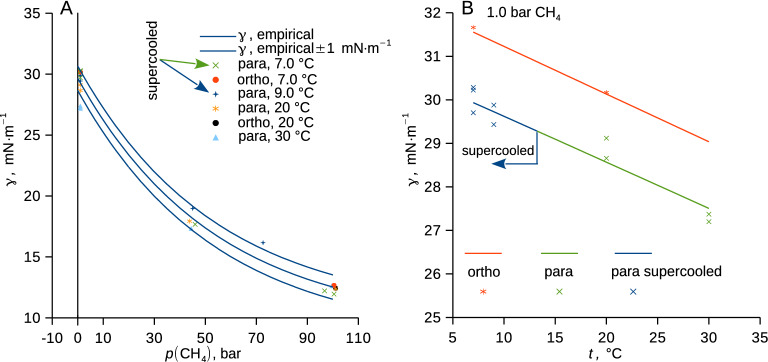
(**A**) Surface tension of *p*-xylene (*p*-C_8_D_10_) and *o*-xylene (*o*-C_8_D_10_) plotted against methane pressure and (**B**) against temperature for a fixed (atmospheric) pressure. Typical uncertainty of surface tension due to random errors (*u*_r_, cover factor 2) is 1–2 mN⋅m^−1^; experimental data are in Table S3 in SI. Curves represent guides for the eye (**A**) and correlations for protium-based xylenes taken from the database^12^; the blue part of the curve is the extrapolation for the supercooled liquid at 1.0 bar (**B**).

**Figure 5:**
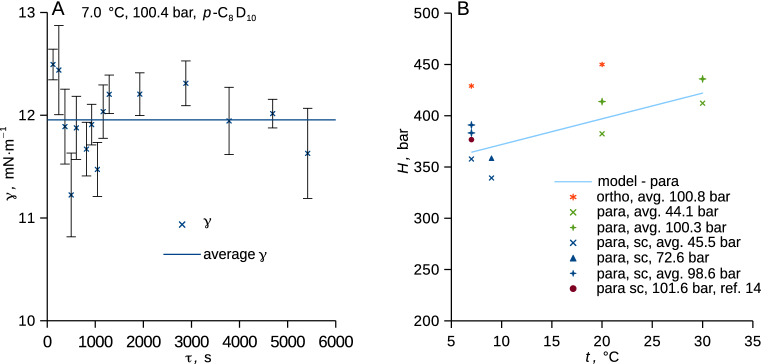
(**A**) Surface tension as a function of time upon exposure to pressurized methane, error bars show uncertainty due to random errors calculated using Bonferroni method^57^, line shows the average. (**B**) Henry’s law constant, see Eq. (5), of methane in the studied deuterated xylenes is shown together with the temperature dependence of Henry’s the constant for all data (this work) for perdeuterated *p*-xylene with the enthalpy of dissolution of − 4.5 kJ⋅mol^−1^ and datum calculated based on the literature^14^ using Eq. (5); sc abbreviates supercooled.

The surface tension of the binary methane solutions with perdeuterated *p*-xylene (*p*-C_8_D_10_) and *o*-xylene (*o*-C_8_D_10_) showed a mild dependence on temperature and a strong dependence on (methane) pressure that followed one master trend irrespective of the supercooling and of the actual xylene isomer (Fig. 4, Table S3 in SI). Surface tension measured for the studied perdeuterated xylenes saturated with methane at 1.0 bar followed the correlations from the database for the pure protium-based xylenes^12^. Interestingly, no influence of the ongoing methane diffusion through the phase interface (Fig. 5A) on the surface tension was detected within the experimental uncertainty. Neither the liquid supercooling nor the actual isomer form of xylene (*o*- and *p*-) thus measurably influenced the methane adsorption on the phase interface, which appears insensitive to the concentration gradient in the liquid. Thus, the measurement of interfacial tension among liquid and gas (or supercritical fluid) does not apparently necessitate reaching the phase equilibrium.

Methane solubility in the xylene phase at the interface was expressed using Henry’s law

5 $$p_{{{\text{CH}}4}} = x_{{{\text{CH}}4}} \cdot H$$

in which *H* is the Henry’s law constant; see^61^ for more discussion. The Henry’s constant for methane and *p*-xylene showed mild dependences on pressure and temperature, no influence of the liquid supercooling was observed to within the experimental uncertainty (Fig. 5B, Table S4 in SI, rel. *u*_c_($H$) ≈ 15%). Our data differed by about 4% from the only available literature datum^14^ for the Henry’s constant of methane in its solution with *p*-xylene (*p*-C_8_H_10_) at relevant conditions. By seeing literature data for supercooled water solutions of volatile compounds^10^, it can be expected that a higher Henry’s constant would be observed for more supercooled solutions of methane and *p*-xylene (*p*-C_8_H_10_).

Henry’s constant of methane in *o*-xylene (*o*-C_8_D_10_) was by about 59 bar higher than that in *p*-xylene (*p*-C_8_D_10_). A similar dependence of the Henry’s constant on the xylene isomerism was reported in the literature^62^ for several hydrocarbon gases dissolved in *o*-xylene (*o*-C_8_H_10_) and in *p*-xylene (*p*-C_8_H_10_).

### Diffusivity and partial molar volume of methane

**Figure 6:**
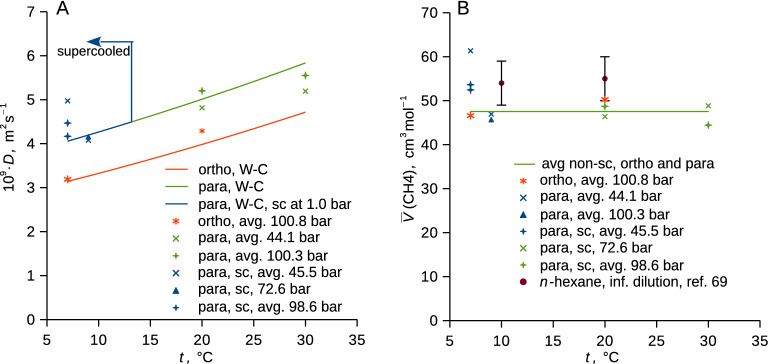
(**A**) Diffusivity and (**B**) partial molar volume of methane for its binary solutions with perdeuterated *p*-xylene (*p-*C_8_D_10_) and *o*-xylene (*o-*C_8_D_10_). Typical uncertainties of diffusivity and partial molar volume due to random error (*u*_r_, cover factor 2) are 0.3·10^–9^ m^2^ s^−1^ and 7 cm^3^ mol^−1^, respectively. Curves in (**A**) represent the Wilke–Chang model^61,63^ with parameters from the database^12^ (viscosity was temperature-extrapolated in the case of *p*-xylene supercooled at 1.0 bar) and with the association factors adjusted to 1.5. Green line in (**B**) shows mean over all non-supercooled systems (this work), literature data for the partial molar volume of methane in *n*-hexane at infinite dilution are shown^64^. Experimental data are listed in Table S4 in SI, sc abbreviates supercooled.

Diffusivity and partial molar volume of methane both showed positive deviations from the master trends due to the supercooling of *p*-xylene, while no such deviations were found for control experiments with *o*-xylene that becomes supercooled at much lower temperatures (Fig. 6, Table S4 in SI). The higher deviations of both quantities were observed at the lowest explored pressure and temperature (average 44.8 bar, 7.0 °C). This suggests that *i*) supercooling of liquid *p*-xylene presumably leads to the molecular-level heterogeneity that facilitates diffusion of methane and reduces the free volume accessible to the methane molecules, *ii*) the dissolved methane disrupts this heterogeneity.

## Conclusions

The supercooling of liquid mixtures of *p*-xylene (*p*-C_8_D_10_) and methane (CH_4_) led to the peculiar increase of the methane partial molar volume and diffusivity under pressures relevant to the *p*-xylene freeze-out in the production of liquefied natural gas (7.0–30.0 °C, 1.0–101.1 bar). Thus, methane diffused more readily in the supercooled solutions, which also showed higher swelling than the normal ones. Systems involving *o*-xylene (*o*-C_8_D_10_) and not showing supercooling were studied for reference. Surface tension was influenced by temperature and pressure and was sensitive neither to the liquid supercooling nor to the isomerism of xylene (ortho, para), no impact of supercooling on the methane solubility was discerned. Our inherently non-tactile neutron imaging method enabled to observe the supercooled liquid bodies and to derive information on their composition and shape at a high spatial resolution (pixel size 20.3 μm). The provision of experimental data at conditions inherently relevant to applications can be fruitful for the community studying supercooled liquids on the molecular level.

## Materials and methods

The neutron imaging experiments were conducted at the NEUTRA beamline^65^ at Paul Scherrer Institute at the measuring position No. 2 (L/D = 365) by MIDI-box detector system using a 30 µm-thick Gd_2_O_2_S:Tb scintillator screen and a sCMOS camera (Andor Neo) fitted with a 100-mm objective (Zeiss Makro-Planar). This experimental arrangement yielded the image of 2560 (W) × 2160 (H) pixels in size with an isotropic pixel pitch of 20.3 µm. The mean distance of the sample to the detector has been equal to 23 mm. The spatial resolution of the resulting images is therefore estimated to be better than 80 µm (based on the intrinsic detector resolution and the beam geometrical unsharpness).The acquisition scheme of the neutron radiographies consisted of several (usually seven) series of 50 images each of the 10 s acquisition time for each investigated system. The raw data were subject to the open beam and the black body corrections^66,67^. For the evaluation of the data from the first two respective series, ten data points were provided as an average of 10 images having the respective time stamp of the average time of the respective 10 images; for the latter series, the entire 50 images were averaged into a single data point having the time stamp of the average of the 50 images.

The experimental setup was described in our previous study^44^ and consisted of a pair of equivalent axially symmetric titanium measuring cells from which one was used for this study were placed in a duralumin block maintained at a constant temperature to within ± 0.2 °C using a Julabo F12-MA water circulator, the temperature was measured using a platinum resistance thermometer (Greissinger GMH 3710), the setup was placed in a duralumin box purged with nitrogen to avoid moisture condensation on the outer parts of the measuring setup. The cells were rinsed with acetone, vacuumed (< 0.01 Pa, Leybold D4B), and twice washed with the fresh sample liquid prior to the filling. One cell contained *p*-xylene (*p-*C_8_D_10_), the other *o*-xylene (*o-*C_8_D_10_) or a heterogeneous system consisting of water (the bottom phase) and *p-*xylene (*p-*C_8_D_10_); data for the latter system will be published elsewhere. Chemicals and gases were used as obtained and are listed in Table 1. The interior of the apparatus was then purged, and the liquids were separately bubbled first with nitrogen and second with methane at atmospheric pressure. Experiments included the steep change of methane pressure from atmospheric to a fixed value, which was maintained constant using a pressure reducer RSD 1 (Siad) to within 0.2 bar and sensed using a PXM409-175BAV (Omega) with a DP41-B control unit (Omega). Experiments were terminated by bubbling the liquid with methane at atmospheric pressure, which was done at 15.0 °C in the case of experiments below the normal melting point of *p*-xylene.

**Table 1:** Used gases and chemicals, initial purity as in the certificate of analysis by the supplier unless indicated otherwise.

Chemical	Supplier, initial purity
Methane (CH_4_)	PanGas, 4.5, CAS 74-82-8
Nitrogen (N_2_)	Messer, 4.0, CAS 7727-37-9
*p*-xylene (*p*-C_8_D_10_)	Armar, 99.59 atom-% D, (> 99.9 wt. %^#^), CAS 41051-88-1
*o*-xylene (*o*-C_8_D_10_)	Armar, 99.52 atom-% D, (> 99.9 wt. %^#^), CAS 56004-61-6
Acetone	Penta, > 99.9 wt.%

^#^Chemical purity was not declared by the supplier and was determined using a GC–MS (Clarus 500, Perkin Elmer) with a capillary column containing Elite WAX ETR stationary phase (Perkin Elmer), value represents purity with respect to other C_8_ aromatic hydrocarbons.

The steep change of methane pressure in the cell inherently causes a shift of the melting temperature of the initially pure liquid *p*-xylene (*p*-C_8_D_10_), which then changes as methane diffuses into the liquid. The degree of supercooling thus changes as methane diffuses through the liquid (Fig. 1 and Table S3 in SI). The melting temperature of perdeuterated *p*-xylene (*p*-C_8_D_10_) and *p*-xylene (*p*-C_8_H_10_) were measured by immersing two ampules with the solidified compounds at atmospheric pressure (rest was air) into the water bath of the Julabo F12-MA water circulator, the temperature was measured using a platinum resistance thermometer (Greissinger GMH 3710). The normal (at 1.0 bar) melting temperature of perdeuterated *p*-xylene (*p*-C_8_D_10_) was 14.1 ± 0.2 °C and that of *p*-xylene (*p*-C_8_H_10_) was 13.3 ± 0.2 °C; the latter agrees with the value from the literature for *p*-xylene (*p*-C_8_H_10_), 13.25 °C^12^. Hence, the melting temperatures of perdeuterated *p*-xylene (*p*-C_8_D_10_) were considered to be by higher 0.8 °C than those for *p*-xylene (*p*-C_8_H_10_)^14^ also in the case of the solutions with methane. The pressure dependence of the melting point of pure *p*-xylene (*p*-C_8_H_10_) was taken from the literature^68^, see Table S3 in SI for details.

## Supplementary Information

Supplementary Information.

## Data Availability

Experimental data are listed in Supplementary Information.
