# Supplementary material for: Absorption of pressurized methane in normal and supercooled p-xylene revealed via high-resolution neutron imaging
Source: Sci Rep. 2023 Jan 4;13:136. doi: 10.1038/s41598-022-27142-6 (PMC9812975; doi:10.1038/s41598-022-27142-6)
Supplement: Supplementary file 1 — Supplementary Information. [file 41598_2022_27142_MOESM1_ESM.docx]

**Supplementary Information**

**Absorption of pressurized methane in normal and supercooled *p*‑xylene revealed via high-resolution neutron imaging**

*Ondřej Vopička^1, *^, Tereza-Markéta Durďáková^1^, Petr Číhal^1^, Pierre Boillat^2,3^, Pavel Trtík^2,*^*

^1^ Department of Physical Chemistry, University of Chemistry and Technology, Prague, Technická 5, 166 28 Prague 6, Czech Republic

^2^ Laboratory for Neutron Scattering and Imaging, Paul Scherrer Institut, 5232 Villigen PSI, Switzerland

^3^ Electrochemistry Laboratory, Paul Scherrer Institut, 5232 Villigen PSI, Switzerland

^*^ Corresponding authors: [ondrej.vopicka@vscht.cz](mailto:ondrej.vopicka@vscht.cz), [pavel.trtik@psi.ch](mailto:pavel.trtik@psi.ch)

The mole concentrations of methane in the gas and supercritical fluid phase were calculated using the Peng-Robinson equation of state ^1^ with parameters (*T*c = 190.564 K, *p*_c_ = 45.99 bar, ω = 0.0115478) taken from the database ^2^. The mole concentrations of the perdeuterated xylenes in the pure liquid were calculated from the known PVT behavior of pure *p*- and *o*-xylene (*p*- and *o*-C_8_H_10_) ^3^ by assuming that the molar volume of the deuterated and protium-based chemicals are equal. The average cross-section [σ, see Eq. (1)] yielded 99 barn for the perdeuterated xylenes and 192 barn for methane over all inspected conditions (Table S1). Importantly, these values held also for supercooled perdeuterated *p*‑xylene (*p*‑C_8_D_10_), thus indicating that solidification did not occur, and density calculated by extrapolating from the region of normal liquid provided a meaningful approximation.

Table S1. Density and cross-section for the pure studied compounds under explored conditions. *u*(*t*) = 0.2 °C, *u*(*p*) = 0.2 bar. Contributions to the uncertainties of the densities are listed in Tab. S2, *u*_r_(C_8_D_10_) = 4 barn, *u*_c_(C_8_D_10_) = 0.5 barn, *u*_r_(CH_4_) = 7 barn, *u*_c_(CH_4_) = 15 barn.

| *t*, °C | *p*, bar | (*p*‑C_8_D_10_), g⋅cm^-3^ | (*o*‑C_8_D_10_), g⋅cm^-3^ | (CH_4_), g⋅cm^-3^ | σ(*p*‑C_8_D_10_), barn | σ(*o*‑C_8_D_10_), barn | σ(CH_4_), barn |
| --- | --- | --- | --- | --- | --- | --- | --- |
| 7.0 | 46.0 | 0.958 |  | 0.036 | 97 |  | 192 |
| 7.0 | 96.7 | 0.962 |  | 0.084 | 98 |  | 193 |
| 7.0 | 100.4 | 0.962 | 0.981 | 0.088 | 98 | 98 | 191 |
| 9.0 | 45.0 | 0.956 |  | 0.035 | 99 |  | 193 |
| 9.0 | 72.6 | 0.958 |  | 0.060 | 100 |  | 195 |
| 20.0 | 43.8 | 0.946 |  | 0.032 | 100 |  | 194 |
| 20.0 | 101.1 | 0.950 | 0.970 | 0.081 | 98 | 97 | 191 |
| 30.0 | 45.5 | 0.937 |  | 0.032 | 103 |  | 184 |
| 30.0 | 99.6 | 0.941 |  | 0.075 | 100 |  | 191 |

Uncertainty due to the random errors (*u*_r_ = 2⋅**σ**_r_, cover factor 2, confidence interval 95 %) was calculated using the Bonferroni method ^4^ in the cases of fitting Eq. (2), Eq. (4) and a straight line (see Fig. 2A) to experimental data. In the other cases, *u*_r_ was calculated based on repeated experiments, **σ** is the sample variance.

The combined systematic uncertainty (*u*_c_) was calculated using the uncertainty propagation law ^5^. For surface tension, this law takes the form

$$\begin{aligned} \left( \frac{u_{c}}{2} \right)^{2}=\boldsymbol{\sigma}_{c}^{2}=\left( \frac{\partial}{\partial t} \right)^{2}\cdot\boldsymbol{\sigma}^{2}\left( t \right)+\left( \frac{\partial}{\partial r} \right)_{r=\frac{R}{2}}^{2}\cdot\boldsymbol{\sigma}^{2}\left( R \right)+\left( \frac{\partial}{\partial g} \right)_{r=\frac{R}{2}}^{2}\cdot\boldsymbol{\sigma}^{2}\left( g \right)+\left( \frac{\partial}{\partial} \right)_{r=\frac{R}{2}}^{2}\cdot\boldsymbol{\sigma}^{2}\left( \right)\#\left( S1 \right) \end{aligned}$$

The derivatives of surface tension with respect to *r*, *g* and were derived based on Eq. (2) at *r* = *R*/2 by assuming, for the purpose of this uncertainty estimation, the spherical shape of the interface and complete wetting. The derivative of surface tension with respect to temperature was assessed using data shown in Fig. 4B in the main text (atmospheric pressure). The uncertainties of the variables in Eq. (S1) are shown in Tables S2 and S3, the resulting combined systematic uncertainty of surface tension for the experiments at atmospheric pressure is *u*_c_(γ) ≈ 0.2 mN⋅m^-1^. The systematic uncertainty predicted using Eq. (S1) is lower than the uncertainty due to random errors (*u*_r_, see Table S3).

Table S2. Uncertainties of parameters in Eq. (S1), *u* = 2⋅**σ**. Contributions to the uncertainty of density were summed up to assess *u*(Δρ).

| Quantity | Uncertainty, *u* | Comment to the assessment |
| --- | --- | --- |
| *t* | 0.2 K | This work |
| *R* | 0.1 mm | Set equal to the uncertainty of cell diameter ^6^ |
| *g* | 0.0007 m⋅s^-2^ | Set to the difference for gravity zones 1 and 1-2 ^7^ |
| *ρ*(EOS-CH_4_) | 0.001 g⋅cm^-3^ | Density difference for methane at 20 °C and 45 bar among the Peng-Robinson EOS ^1^ and the Setzmann-Wagner EOS ^8^. |
| *ρ*(evap-CH_4_) | 0.002 g⋅cm^-3^ | Error due to the *p*-xylene evaporation into methane. The estimate is based on the reported concentration of methane (99.52 mol.% CH_4_) in the saturated methane phase for system methane and *m*‑xylene (*m*‑C_8_H_10_) at 115.8 bar and 310.9 K ^9^. |
| *ρ*(*p*-C_8_H_10_) | 0.00035 g⋅cm^-3^ | Average deviation of the density model for liquid *p*‑xylene (*p*‑C_8_H_10_) for data ranging 288.18 to 548.15 K, RMSD in Table 4 in reference ^3^. |
| *ρ*(*p*-C_8_D_10_) | 0.003 g⋅cm^-3^ | Expected non-constancy of the molar volume of *p*‑xylene (*p*‑C_8_H_10_ and *p*‑C_8_D_10_) due to deuteration provided that the effect is comparable to that in benzene at 20 °C and 100 bar ^3^. |

The melting point of pure protium-based *p*-xylene at atmospheric pressure was taken from the database ^2^. The melting point of pure protium-based *p*-xylene at elevated pressure was assessed using the Simon equation fitted to a dataset of melting temperatures at elevated pressures ^10^ and at atmospheric pressure ^2^, thus yielding

$$\begin{aligned} p_{m}=-4495+0.1181\cdot T_{m}^{1.8644}\#\left( S2 \right) \end{aligned}$$

where *p*_m_ and *T*_m_ are melting pressure in bar and melting temperature in K, respectively. In all cases, the melting temperature of perdeuterated *p*-xylene (*p*-C_8_D_10_) was assumed to be by 0.8 °C higher than that for the protium-based *p*-xylene (*p*-C_8_H_10_) as this temperature difference was observed by us at atmospheric pressure (see main text). The i*nitial supercooling* was thus calculated for the pure *p*-xylene (*p*-C_8_D_10_) exposed to elevated pressure. The melting point of the solutions of methane and protium-based *p*-xylene (*p*-C_8_H_10_) was taken from the literature ^11^ and used to calculate the s*upercooling at equilibrium*, for the solutions of methane (CH_4_) with *p*‑xylene (*p*‑C_8_D_10_); see Table S3.

Table S3 tension, contact angle, degree of supercooling. The density of the saturated binary liquid solutions (CH_4_ with *p*-C_8_D_10_; CH_4_ with *o*-C_8_D_10_) was derived using Eq. (2), degree of supercooling was derived as described above. For all inspected conditions, we found contact angle θ = (14 ± 2) °, the uncertainties of the density of the solutions at elevated pressures due are *u*_r_(${}^{\mathrm{liq}}$) ≈ *u*_c_(${}^{\mathrm{liq}}$) ≈ 0.02 g⋅cm^-3^. *Density for liquids at 1.0 bar of methane was calculated as density of pure liquid using literature models of PVT behavior ^3^ recalculated to the perdeuterated xylenes, see Table S2 for expected uncertainty.

| Liquid systems formed by methane (CH_4_) and perdeuterated *p*-xylene (*p*‑C_8_D_10_) | | | | | | |
| --- | --- | --- | --- | --- | --- | --- |
| *t*, °C | *p*, bar | $\gamma$, mN⋅m^-1^ | *u*_r_$(\gamma)$, mN⋅m^-1^ | ${}^{\mathrm{liq}}$, g⋅cm^-3^ | *Initial supercooling*, °C | *Supercooling at equilibrium*, °C |
| 7.0 | 46.0 | 18 | 1 | 0.91 | 8.6 | 4.1 |
| 7.0 | 96.7 | 12 | 1 | 0.87 | 10.3 | 1.0 |
| 7.0 | 100.4 | 12 | 1 | 0.88 | 10.4 | 0.8 |
| 7.0 | 1.0 | 30 | 2 | 0.955* | 7.1 | 7.1 |
| 7.0 | 1.0 | 30 | 1 | 0.955* | 7.1 | 7.1 |
| 7.0 | 1.0 | 30 | 2 | 0.955* | 7.1 | 7.1 |
| 9.0 | 45.0 | 19 | 1 | 0.92 | 6.5 | 2.2 |
| 9.0 | 72.6 | 16 | 1 | 0.90 | 7.5 | 0.4 |
| 9.0 | 1.0 | 30 | 1 | 0.953* | 5.1 | 5.1 |
| 9.0 | 1.0 | 29 | 1 | 0.953* | 5.1 | 5.1 |
| 20.0 | 43.8 | 18 | 1 | 0.92 | NA | NA |
| 20.0 | 101.1 | 12 | 1 | 0.88 | NA | NA |
| 20.0 | 1.0 | 29 | 2 | 0.943* | NA | NA |
| 20.0 | 1.0 | 29 | 1 | 0.943* | NA | NA |
| 30.0 | 45.5 | 17 | 1 | 0.90 | NA | NA |
| 30.0 | 99.6 | 13 | 1 | 0.88 | NA | NA |
| 30.0 | 1.0 | 27 | 1 | 0.933* | NA | NA |
| 30.0 | 1.0 | 27 | 1 | 0.933* | NA | NA |
| Liquid systems formed by methane (CH_4_) and perdeuterated *o*‑xylene (*o*‑C_8_D_10_) | | | | | | |
| 7.0 | 100.4 | 13 | 1 | 0.91 | NA | NA |
| 7.0 | 1.0 | 32 | 2 | 0.963* | NA | NA |
| 20.0 | 101.1 | 12 | 1 | 0.90 | NA | NA |
| 7.0 | 1.0 | 30 | 2 | 0.975* | NA | NA |

Experimental data for diffusivity and partial molar volume of methane for the studied systems are listed in Table S4 together with the molar fraction of methane in the solutions at absorption equilibrium, which compares well to the literature datum ^11^.

Table S4. Experimental data on methane solubility, diffusivity, and partial molar volume in *p*‑xylene (*p*‑C_8_H_10_). ^#^Equilibrium composition of liquid composed from protium-based *p*‑xylene (*p*‑C_8_H_10_) and methane at 7.0 °C and 101.6 bar from the literature ^11^. Uncertainty due to random errors (*u*_r_, cover factor 2) is listed. Relative combined systematic uncertainties are: rel. *u*_c_($\bar{V}_{\mathrm{CH}_{4}}$) ≈ 10 %, rel. *u*_c_($x_{\mathrm{CH}_{4}}$) ≈ 15 %.

| Systems from methane (CH_4_) and perdeuterated *p*-xylene (*p*‑C_8_D_10_) | | | | | | | |
| --- | --- | --- | --- | --- | --- | --- | --- |
| *t*, °C | *p*, bar | $x_{\mathrm{CH}_{4}}$ | *u*_r_($x_{\mathrm{CH}_{4}}$) | 10^9^⋅*D*, m^2^s^-1^ | 10^9^⋅ *u*_r_(*D*, m^2^s^-1^) | $\bar{V}_{\mathrm{CH}_{4}}$, cm^3^mol^-1^ | *u*_r_$(\bar{V}_{\mathrm{CH}_{4}})$, cm^3^mol^-1^ |
| 7.0 | 46.0 | 0.13 | 0.03 | 5.0 | 0.3 | 61 | 6 |
| 7.0 | 96.7 | 0.25 | 0.04 | 4.5 | 0.2 | 54 | 2 |
| 7.0 | 100.4 | 0.26  0.2697^#^ | 0.05 | 4.2 | 0.2 | 52 | 4 |
| 9.0 | 45.0 | 0.13 | 0.05 | 4.1 | 0.3 | 47 | 7 |
| 9.0 | 72.6 | 0.20 | 0.08 | 4.2 | 0.2 | 46 | 8 |
| 20.0 | 43.8 | 0.11 | 0.04 | 4.8 | 0.4 | 46 | 10 |
| 20.0 | 101.1 | 0.24 | 0.05 | 5.5 | 0.2 | 49 | 2 |
| 30.0 | 45.5 | 0.11 | 0.04 | 5.2 | 0.4 | 49 | 3 |
| 30.0 | 99.6 | 0.23 | 0.05 | 5.6 | 0.2 | 44 | 4 |
| Systems from methane (CH_4_) and perdeuterated *o*‑xylene (*p*‑C_8_D_10_) | | | | | | | |
| 7.0 | 100.4 | 0.23 | 0.04 | 3.2 | 0.2 | 47 | 3 |
| 20.0 | 101.1 | 0.22 | 0.04 | 4.5 | 0.2 | 50 | 8 |

**REFERENCES**

1 Peng, D.-Y. & Robinson, D. B. A New Two-Constant Equation of State. *Industrial & Engineering Chemistry Fundamentals* **15**, 59-64, doi:10.1021/i160057a011 (1976).

2 Design Institute for Physical Properties, Sponsored by AIChE, DIPPR Project 801 - Full Version, Retrieved from <https://app.knovel.com/hotlink/toc/id:kpDIPPRPF7/dippr-project-801-full/dippr-project-801-full> (January 30, 2020).

3 Cibulka, I. & Takagi, T. P−ρ−T Data of Liquids:  Summarization and Evaluation. 5. Aromatic Hydrocarbons. *Journal of Chemical & Engineering Data* **44**, 411-429, doi:10.1021/je980278v (1999).

4 Seber, G. A. F. & Wild, C. J. *Nonlinear Regression*. (John Wiley & Sons, Inc., 2003).

5 *Evaluation of measurement data — Guide to the expression of uncertainty in measurement* (JCGM, 2008).

6 Vopička, O. *et al.* One-pot neutron imaging of surface phenomena, swelling and diffusion during methane absorption in ethanol and n-decane under high pressure. *PLOS ONE* **15**, e0238470, doi:10.1371/journal.pone.0238470 (2020).

7 *Swiss Gravity Zones* <<https://www.metas.ch/metas/en/home/dok/gravitationszonen.html>>

8 Setzmann, U. & Wagner, W. A New Equation of State and Tables of Thermodynamic Properties for Methane Covering the Range from the Melting Line to 625 K at Pressures up to 1000 MPa. *Journal of Physical and Chemical Reference Data* **20**, 1061-1155, doi:10.1063/1.555898 (1991).

9 Ng, H. J., Huang, S. S. S. & Robinson, D. B. Equilibrium phase properties of selected m-xylene binary systems. m-Xylene-methane and m-xylene carbon dioxide. *Journal of Chemical & Engineering Data* **27**, 119-122, doi:10.1021/je00028a004 (1982).

10 Nagaoka, K. & Makita, T. Effect of pressure on the solid-liquid phase equilibria of (carbon tetrachloride + p-xylene) and (carbon tetrachloride+benzene) systems. *International Journal of Thermophysics* **9**, 535-545, doi:10.1007/BF00503152 (1988).

11 Siahvashi, A. *et al.* Solubility of p-xylene in methane and ethane and implications for freeze-out at LNG conditions. *Experimental Thermal and Fluid Science* **105**, 47-57, doi:10.1016/j.expthermflusci.2019.03.010 (2019).
